# Clinical, Histopathological and Therapeutical Analysis of Inferior Eyelid Basal Cell Carcinomas

**Published:** 2014

**Authors:** M Totir, C Alexandrescu, R Pirvulescu, S Gradinaru, M Costache

**Affiliations:** * University Emergency Hospital Bucharest – Department of Clinical Ophthalmology; ** University of Medicine and Pharmacy “Carol Davila” Bucharest, Romania – Ophthalmology Department; *** Regina Maria Private Health Care Network – Ophthalmology Department; **** University Emergency Hospital Bucharest – Department of Anatomo-pathology

**Keywords:** basal cell carcinoma, inferior eyelid, histology, frozen section

## Abstract

**Rationale:** Eyelids are very susceptible area for non-melanoma skin cancers; among that, basal cell carcinoma has the highest incidence (almost 90% of malignant eyelid tumors) and 50-60% of eyelid basal cell carcinomas appear on inferior eyelid.

**Objective:** To analyze clinical features of inferior eyelid basal cell carcinoma and to determine the efficacy of surgical treatment with frozen sectioncontrolled margins and methods of primary reconstruction of defects.

**Methods:** A review of medical records of cases with primary inferior eyelid basal cell carcinoma treated by surgical excision with urgent histopathology controlled margins by FS technique, doubled by paraffin examination from October 2011 to October 2014. After histopathology confirmation of tumor free margins, proper inferior eyelid reconstruction was performed.

**Results:** The review resulted in 36 patients with 36 lesions analyzed by clinical, histopatological and therapeuticalaspectswith a mean follow-up of 20 months. All lesions were primary BCC affecting inferior eyelid. There were no recurrence in the follow-up period. Inferior eyelid reconstruction techniques were direct closure for small defects and complex techniques for defects more than one third of eyelid length.

**Discussion:** Appropriate eyelid examination is mandatory in any routine ophthalmic check-up. Clinical signs suggestive of BCC should be familiar to ophthalmologist in order to have an early diagnosis and treatment for these tumors. Surgical treatment with FS controlled excision followed by eyelid reconstruction is an efficient treatment for inferior eyelid BCC.

**Abbreviations:** basal cell carcinoma (BCC); frozen section (FS);Mohs micrographic surgery (MMS).

## Introduction

The incidence of non-melanoma skin cancer has rapidly increased in the last years .Five to ten percent of all skin cancers occur on the eyelid. BCC is the most frequent skin cancer in periocular region, accounting for 90% of eyelid tumors. [**1**] BCC has a low metastatic potential but it is locally destructive and invasive to deeper structures. Left untreated, BCC can have not only serious aesthetic but also functional meanings because of invasion to the orbit and craniofacial structures.

Reviewing published reports regarding the treatment of basal cell eyelid tumors we see that there are described many options of non-surgical or surgical treatment. The non-surgical treatment includes cryotherapy, photodynamic therapy, radiation, topical 5-fluorouracil, topical imiquimod, but non-surgical management has the disadvantage of absence of histopathologic confirmation for correct diagnosis of tumor type or for complete tumor eradication. [**2**] Surgical treatment offers best curability rates and remains gold standard for BCC. Surgical resections used are excision with predetermined margins, FS controlled excision and Mohs micrographic surgery (MMS) with cure rates between 80 and 99%. [**3**,**4**,**5**]

Excisional biopsy with predetermined margin is an easy and popular technique but there is a lack of consensus regarding the need of tissue to be excised (2-5mm margins for periocular BCC). Hsuanet al reported that for a 2mm margin excision 10 patients out of 55 needed reexcision for free tumor margin, while Hamada et al found clear margins for 84% of lesions excised at 4mm margins;still because standardization of predetermined margins is not possible and in the eyelids the amount of tissue excised is essential, the need for histology clear margin confirmation remains an important issue. [**6**,**7**]

MMS is a surgical technique that allows precise microscopic control of the margins with the most effective preservation of normal tissue (tissue is excised in horizontal layers that provide a three-dimensional mapping of the excised tumour). In 1986, Mohs showed a 99,4% cure rate at 5 years for primary periocular BCC in 1124 cases using MMS [**8**]. In the light of these numbers, there are practitioners that support the idea that MMS should be the first line treatment for BCC and is mandatory for recurrences and tumours with high chance of recurrence (medial canthus localization), tumours with lacrimal extension andtumours larger than 3cm.[**9**, **10**,**11**]. MMS has disadvantages like cost, prolonged time of intervention and necessity of a specially trained personnel because histologic preparation and interpretation of microscopic examination requires skill and practice.

In FS-controlled excision, the cutting is done verticallyandprocessed by bread loafing technique, making some authors say that a smaller percent of margins are examined than with Mohs technique; despite that, studies showed that frozen section technique for BCC has comparable curability rates with Mohs surgery [**12**,**13**]

## Methods

This study was conducted in compliance with good clinical practice, institutional review board regulations, informed consent regulations and the principles of Declaration of Helsinki.

We reviewed medical records of patients who underwent full-thickness frozen section controlled excision for primary inferior eyelid basal cell carcinoma involving the eyelid margin, between October 2011 and October 2014.

**Fig. 1:**
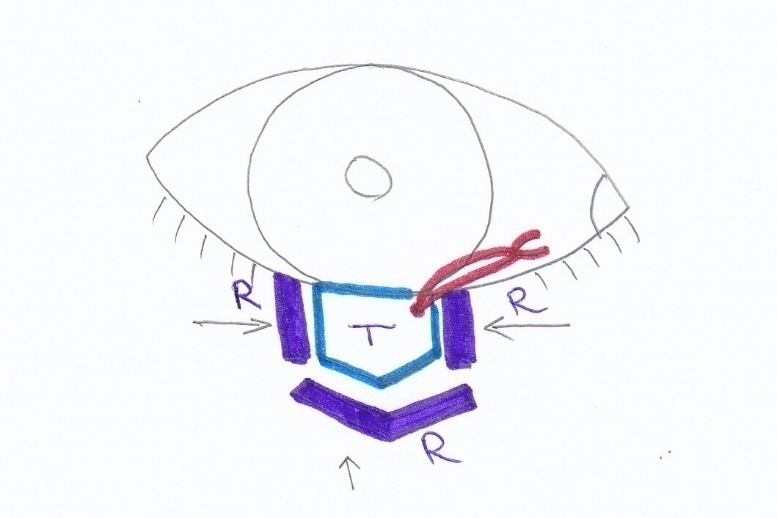
Protocol for full thickness eyelid excision with en face frozen section margin control (arrows show the face that needsto be analyzed)

All patients underwent a complete ophthalmic examination before surgery. We noted personal data: age, gender, risk factors (smoke, ultraviolet exposure, personal and family history of cutaneous malignancies, ultraviolet exposure), clinical data (anatomical location in the lower eyelid, clinical aspects of the tumor, size based on maximum diameter), histological subtype, reconstruction technique, complications, duration of follow-up. Tumor excision protocol was to remove visible tumor with 1 mm peritumoral apparent healthy tissue and also another 3 reexcision sections of 1 mm width (**Fig. 1**). The tumor specimen is sent for paraffin examination and reexcision sections are sent face-up forimmediate histopathological margin controlby frozen technique at -24 C using hematoxylin-eosin staining. When residual tumor cells were discovered, a new reexcision of 1 mm was performed in the noted area until the examination shows no tumoral cells. The examination is doubled by final paraffin examination.

T – Tumor removed with 1mm apparent healthy tissue and medial red marking suture

R – 1mm reexcision sections

## Results

We reviewed all cases of primary inferior eyelid BCC involving eyelid margin who were treated by full thickness frozen section controlled excision between October 2011 and October 2014 and resulted in 39 lesions in 39 patients; we excluded three cases because of orbital or lacrimal invasion and the rest of 36 were analyzed. 

**Fig. 2:**
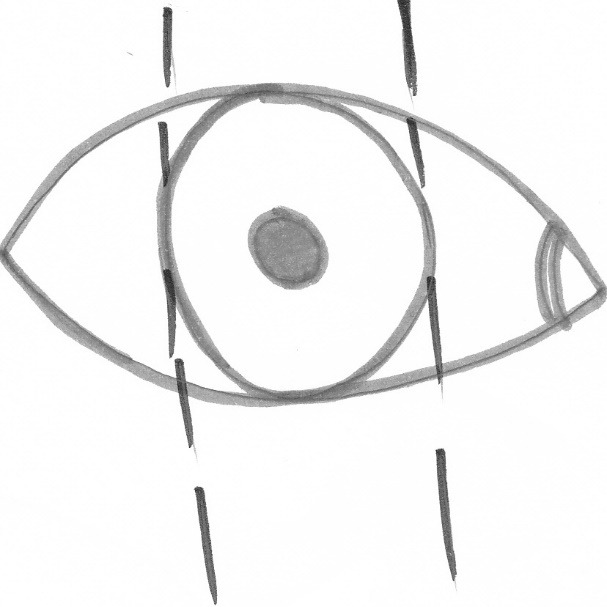
Anatomical locations of inferior eyelid BCC (36 cases)

**Fig. 3:**
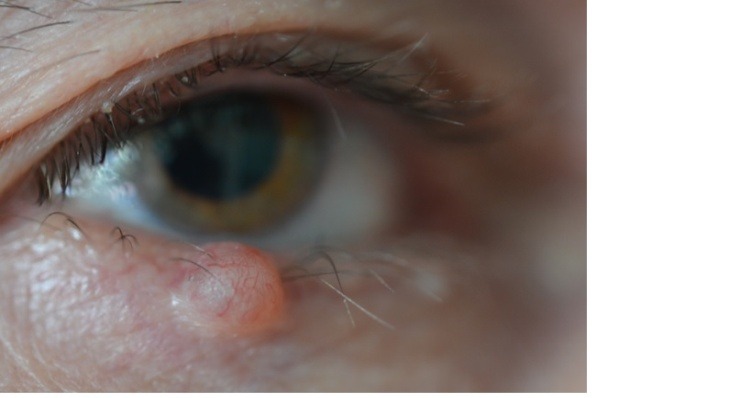
Clinical aspect of cystic BCC

**Fig. 4:**
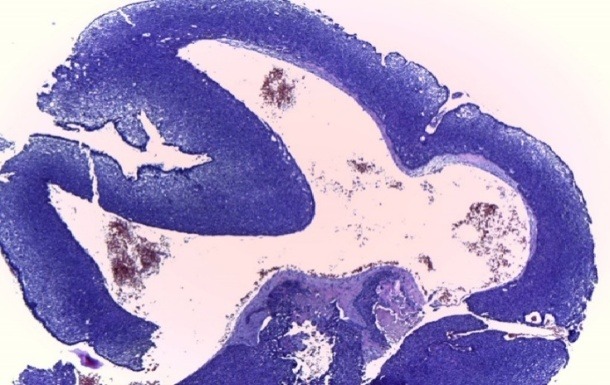
Histopathological aspect of cystic BCC – cystic cavities inside tumoral islands

The demographic data are shown in (**Table 1**). The right eye was involved in 9 patients (25%) and the left eye in 27 (75%).The anatomical distribution on different regions of the inferior eyelid is represented in (**Fig. 2**). The frequent clinical aspects of inferior eyelid BCC: ulceration, induration, localized loss of lashes, lesions with pearly borders, telangiectatic vessels, alteration of normal eyelid architecture made easy the clinical diagnosis of BCC; we had only one patient in which clinical aspect of the lesion was more particular, with a round, well-defined, cystic lesion where a histopathological examination established certitude diagnosis of cystic BCC subtype. (**Fig. 3**, **Fig. 4**). We also noted association of blepharitis in 22,22% of patients and values of Schirmer test between 4-20mm at 5minutes without anesthesia. (**Table 2**) gives information about lesion size, histopathological subtype, and reconstructive techniques. Mean duration of follow-up was 20 months with range between 3 and 36 months. We had no recurrences during the follow-up period. We had only one complication after surgery in a patient who presented with localized trichiazis resolved with localized cilia electrolysis. 

**Table 1:** Demographic data

Cases=36	Number
Gender (male/female)	12/24
Age (y) mean, range	65, 49-83
Urban/rural provenience	15/21

**Table 2:** Inferior eyelid BCC characteristics, histopathology and reconstructive technique

Cases =36	
Lesion size	
mean, range (mm)	7.16 (2.0-15.0)
Histopathological subtype	
Nodular	24
Cystic	1
Superficial	6
Infiltrative	5
Reconstruction technique	
Direct closure	22
Periosteal flap,local advancement flaps	8
Complex techniques for entire eyelid	6

## Discussion

BCC is the most common malignant eyelid tumor and the incidence continues to grow with the increase of hope life expectancy (as older age is a risk factor) and excessive ultraviolet light exposure (especially among young people). [**14**,**15**]. The inferior eyelid continues to be the most common site for periocular BCC, some authors noting an incidence up to 60%.

In our study, the mean age of these patients was 65 years and we did not find exposure to ionized radiation, chemical agents, immunosuppression or predisposing genetic disease in any of our patients. We had only one case with important family medical history in a female with infiltrative BCC 55years old with a sister who died of malignant melanoma, arm localization.

Examining patients and discovering that blepharitis can be an associated local condition to eyelid BCC led to the question if eyelid tumors affecting eyelid margin are influenced by the permanent inflammation caused by blepharitis.

Early diagnosis and correct treatment with complete tumor removal give high curability rate. Bernardini stated that Mohs surgery or complete surgical excision with frozen-section control of the margins offers the lowest tumor-recurrence rate. [**16**] GiordaniResti et al found an overall recurrence rate of 1.8%, with a mean follow-up of 72.4 months (range 30-167) from 110 malignancies concluding that FS excision of eyelid BCCs yields recurrence rates comparable to those of MMS at 5-year follow-up. [**17**] In our series of patient we found no recurrences in the follow-up period, but we need to continue monitor the patients to larger periods of time.

Wu et al found in 1713 consecutive periocular BCC excision specimens that 52.7% involved the lower eyelid and the main histological subtypes identified were nodular (65.7%); our analysis showed nodular histopathological subtype in 66,67% cases and the infiltrative subtype appeared in patients aged less than 60 years old. [**18**]

**Fig. 5:**
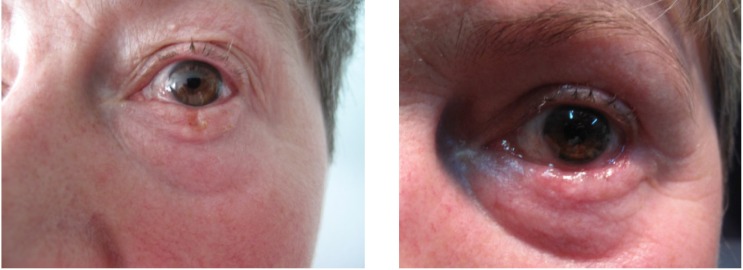
Patient who underwent FS controlled excision and entire inferior eyelid reconstruction (before and after)

We performed inferior eyelid reconstruction by direct suture in the majority of cases but complex reconstruction for entire eyelid was performed in 6 cases with separate anterior and posterior lamellar reconstruction; we used Hughes tarsoconjunctival flap or buccal mucosa and auricular cartilage for the posterior lamella and local flaps usually from the superior eyelid for the anterior lamella with good functional and aesthetic outcome. (**Fig. 5**) shows photos of a patient before and after FS excision and reconstruction of entire eyelid with buccal mucosa, auricular cartilage and superior eyelid flap.

The limitations of our study can be pointed in the relatively small number of cases (compared to studies from multicenter units) followed for a period of time of less than five years, nevertheless we consider important that average lesion size (7.16mm) was bigger than other studies and still we had no recurrence during our follow-up period.

In conclusion, BCC diagnosis should be done early and proper treatment recommended. Surgical treatment with histopathological margin control remains standard gold for the treatment of periocular BCC followed by eyelid reconstructionwith restoration of eyelid structure and function to as near normal possible.

**Disclosure**

None
